# Plain access to justice and the orthodontist’s activity in Brazil: vulnerability in the professional practice in the face of risks of malpractice lawsuits

**DOI:** 10.1590/2177-6709.23.4.088-093.sar

**Published:** 2018

**Authors:** Cleverson Raymundo Sbarzi Guedes, Isabel Cristina Gonçalves Leite, Marcio José da Silva Campos, Sergio Luiz Mota, Matheus Melo Phiton, Robert Willer Farinazzo Vitral

**Affiliations:** 1 Universidade Federal de Juiz de Fora, Faculdade de Direito, Departamento de Direito Público Material (Juiz de Fora/MG, Brazil) Universidade Federal de Juiz de Fora Universidade Federal de Juiz de Fora Faculdade de Direito Departamento de Direito Público Material Juiz de ForaMG Brazil; 2 Universidade Federal de Juiz de Fora, Faculdade de Medicina, Departamento de Saúde Coletiva (Juiz de Fora/MG, Brazil) Universidade Federal de Juiz de Fora Universidade Federal de Juiz de Fora Faculdade de Medicina Departamento de Saúde Coletiva Juiz de ForaMG Brazil; 3 Universidade federal de Juiz de Fora, Faculdade de Odontologia, Departamento de Odontologia Social e Infantil (Juiz de Fora/MG, Brazil) Universidade Federal de Juiz de Fora Universidade federal de Juiz de Fora Faculdade de Odontologia Departamento de Odontologia Social e Infantil Juiz de ForaMG Brazil; 4 Universidade Estadual do Sudoeste da Bahia, Departamento de Saúde (Jequié/BA, Brazil). Universidade Estadual do Sudoeste da Bahia Universidade Estadual do Sudoeste da Bahia Departamento de Saúde JequiéBA Brazil

**Keywords:** Civil responsibility, Validation study, Lawsuits, Compensation

## Abstract

**Objective:**

the present study aimed at evaluating the risks and vulnerability of orthodontists to legal compensation actions and verifying the hypothesis of these health care professionals having little knowledge concerning their rights and obligations as service providers.

**Methods:**

Three groups were formed to participate in a semi-structured interview. The first group had thirteen law professionals, the second group was composed of eleven orthodontists and the third group was made up of nine randomly selected orthodontic patients.

**Results:**

Relevant aspects related to the exercise of the professional activity of orthodontists that influence on the vulnerability of orthodontists in lawsuits were identified. After transcription, reading, and comparing the answers of the interviews, items capable of influencing judicial decisions, from the standpoint of Brazilian Justice Courts, were evaluated.

**Conclusion:**

It was verified that Brazilian orthodontists do not have adequate formation concerning the legal consequences of the exercise of their professional activity. Orthodontists also failed to establish proper contractual relationship, organize orthodontic records, and, most importantly, failed in communicating the risks and the therapeutic processes to patients during all phases of treatment.

## RESUMO

**Objetivo:**

o presente trabalho teve como objetivo apurar os riscos e a vulnerabilidade dos profissionais ortodontistas serem envolvidos em lides judiciais com finalidade indenizatória, além de verificar a hipótese de que esses profissionais possuem pouco conhecimento a respeito de suas obrigações e deveres como prestadores de serviço, bem como dos mecanismos que podem evitar tais demandas judiciais.

**Métodos:**

foram formados três grupos para entrevistas semiestruturadas, compostos por profissionais da área jurídica, profissionais ortodontistas e pacientes ortodônticos: no primeiro grupo, foram entrevistados 13 (treze) profissionais da área jurídica; no segundo grupo de entrevistados, foram abordados 11 (onze) profissionais ortodontistas; para o terceiro grupo de entrevistas, foram sorteados 9 (nove) pacientes em tratamento ortodôntico.

**Resultados:**

foram identificados itens ou domínios relevantes, relacionados ao exercício da atividade profissional dos ortodontistas, que repercutem diretamente em aspectos de vulnerabilidade deles, quando confrontados, objetivamente, com fundamentos de decisões judiciais envolvendo questões indenizatórias em face de tais profissionais da área da saúde. Após o trabalho de transcrição das entrevistas, leitura, compilação e comparação das respostas, passou-se à análise dos temas capazes de influenciar na rotina forense, a partir das tendências doutrinárias e jurisprudenciais encontradas nos Tribunais brasileiros.

**Conclusão:**

verificou-se que os ortodontistas não apresentam formação adequada para o completo entendimento sobre as consequências jurídicas do exercício de sua atividade profissional. Constataram-se, também, falhas por parte do profissional ortodontista, desde o estabelecimento da relação contratual, formação de documentação ortodôntica organizada e, sobretudo, registro, documentação e informação aos pacientes, ou responsáveis legais, em todas as etapas do tratamento.

## INTRODUCTION

Dentists, particularly orthodontists, are potential targets for compensation lawsuits and frequently find themselves unprepared, facing unnecessary risks of legal actions concerning their treatment^1^. Cases involving the specialties of oral maxillofacial surgery and orthodontics, quite similarly, were the most involved specialties in legal actions with the Court of Justice of Rio Grande do Sul (Brazil), between 2007 and 2010.^2^

It is obersved that dentist’s activity, particularly the orthodontist, involves a great debate concerning compulsory aspects included on the signing of the orthodontic treatment contract.^3^ This results from eventual or future discussion about reparation for harm done, from the aspects of objective or subjective responsibility,^4^ as well as the characterization of the professional activity, legally, as an obligation of means or results,^5^ to the influence of the Consumer Defense Code as an instrument of regulation of the established relationship between patient and health professional.^6^^-^^9^

Orthodontists’ liability, as well as other health professionals, is based on the tripod of criminal, ethical and civil liabilities.^10^^,^^11^ Unlike criminal responsibility, of little occurrence in dentistry,^12^^,^^13^ civil responsibility in the Brazilian law system provided fertile ground for its evolution, from the Civil Code of 1916 to its substitution in 2002, as well as the Consumer Defense Code^14^ in 1990 and, more recently, validity of the New Civil Process Code.^15^^,^^16^ Civil responsibility consists in determining the obligation for reparation of harm caused to others, and it is based, therefore, on represented assumptions by a legal action (or right violation) and harm (material or moral) associated with a casual connection.^17^^-^^19^

After a number of years of exercise of citizenship, especially after the Federal Constitution of 1988, every citizen has been able to experience major access to justice and health service.^20^ In view of the bankruptcy of the state machine, it can be noted a judicialization of health, at the same time a great demand with the organs of the Judiciary Power for the guarantee of rights, both individual and collectively, takes place, revealing the judicial activism as the face of democratic legitimation.^21^ Indeed, all difficulties found by the citizen to access justice have turned into facilities and stimuli to do so, trivializing, in a certain way, the access to justice.^22^

From this assumption, the present study aims at investigating the risks and vulnerabilities of orthodontists being involved in legal compensation actions, as well as verifying the hypothesis that these professionals have little knowledge concerning their obligations and duties as service providers and the mechanisms that could avoid legal demands. 

## MATERIAL AND METHODS

The research project was submitted to and approved by the Ethics and Research Committee of the Federal University of Juiz de Fora, under the protocol number 1.403.552. A written informed consent was signed by all participants of the interviews.

Three groups were formed for the semi-structured interviews: the first group was composed of 13 law professionals (G1), whilst the second and third groups were made up of 11 orthodontists (G2), and 9 orthodontic patients (G3), respectively. 

Group 1 had 13 civil law postgraduate professionals with emphasis on compensation actions. In the semi-structured interviews, questions were asked concerning the participants’ experience and effective acting in demands involving health professionals, particularly dentists, and their preparation to perform comprehensive defense in compensation actions. Questions also addressed the technical preparation and specific knowledge of judges in legal actions involving health professionals, patient-professional relationship and their eventual negative consequences that may lead to legal demands, application of the Consumer Defense Code, technical aspects of the attribution of blame by health professionals, and decisive aspects in the making up of decisions contrary to health professionals in indemnity actions.

In group 2, eleven orthodontists were selected from a nominal data bank. Of these, 8 worked exclusively with orthodontics. In the interviews, inductive and informative questions were addressed to the orthodontists, eventually with explanation about some juridical terms, related to their academic and professional formation, postgraduate education, patient selection, clinical and complementary examinations, patient-orthodontist relationship, orthodontic records, service agreements, patient orientation, control of treatment from the beginning to the end, concern about possible legal actions due to eventual dissatisfaction of the patient, and orientation by a lawyer. 

In group 3, nine orthodontic patients were randomly selected from different orthodontic practices, in different phases of treatment. For patient selection, a name list was voluntarily provided by the interviewed orthodontists, all of which declared not having any legal action against them. For this group, ordinary inductive questions were asked with eventual explanation about juridical terms, emphasizing the choosing criteria of the orthodontist, access to information about cost, time, orthodontic treatment techniques, orthodontist’s demands to start treatment, esthetic or functional concerns, patient-professional relationship during all phases of treatment, attitudes towards the dissatisfaction of the patient with treatment, and the juridical consequences of such possibility. 

The semi-structured interviews were made directly by the researcher after setting appointment directly with the interviewees. All interviews were reserved and recorded by the researcher through previous authorization of the interviewees, and always anonymously.

After the transcription, reading, compilation, and comparison of the answers of the interviews, the themes capable of influencing judicial decisions, from the standpoint of the Brazilian Justice Courts, were evaluated.

## RESULTS

The interviews showed that relevant domains and items related to the exercise of the activity of orthodontist influence directly on the vulnerability of these professionals when confronted with judicial decisions involving compensation suits.

### Patient-orthodontist relationship

In the three groups used in the interviews, a failure of the patient-professional relationship could be observed as a triggering element for legal compensation actions or as a real background for the start of lawsuits. Direct or indirectly, all the interviewees indicated that the lack of straight and transparent conversation between orthodontists and patients would invariably lead to the possibility of malpractice suits. The magistrates stated that in the lawsuits involving health professionals (including particularly physicians and dentists) there was failure in the conversation between the parties. In many instances, conciliation and mediation approaches were necessary to bring the parties together.

Assessment of the difficulty in the patient-professional relationship demonstrated that neither one of the parties had full knowledge of their contractual obligations. Orthodontists did not have complete knowledge about the economic, cultural and social profile of their patients, essential tool to obtain the free informed consent. This instrument is indispensable for the juridical relation that is established between the parties during a significant period of treatment, corresponding to the validity of the contractual relationship between the consumer and the service provider.

### Formation and capacitation of the orthodontist

Interviews demonstrated, notedly between orthodontists and lawyers, that in many circumstances orthodontists run significant risk of lawsuits because of emphatic deficiencies in their formation and capacitation for the exercise of their profession. Occasionally, ethical administrative and judicial processes result in conviction of orthodontists that could have been avoided, if it were not for an inadequate formation or insufficient or deficient capacitation.

### Orthodontists’ awareness about juridical repercussions of their professional obligations

The answers of the interviews demonstrated that there were few graduate schools in Dentistry with Juridical Deontology in their curriculum. Even in postgraduate courses, there is a lack of instruments capable of providing the necessary and essential knowledge for the orthodontist to act even in a preventive manner.

### Contractual relationship for service provider

As a result of the partial unknowing of orthodontists’ obligations during the exercise of their activity, it is important to highlight certain parts of the interview in which the parties involved in an orthodontic treatment hardly speak about the contractual relationship. 

In cases with service agreement contract, a standardized form was used as solution in an attempt for the orthodontist to avoid unpleasant lawsuits. Such alternative has shown to be inadequate, revealing many times that there is not any customization in the contractual questions. That is exactly what can be used against the orthodontist in a judicial demand. 

There are rare cases in which technical orientation is given by a lawyer for the formulation of contracts, distracts and contract clause alterations, a fact stressed in ethical administrative processes in which not even a formalized contract could be found. 

### Organization and maintenance of orthodontic records

Most often, according to magistrates and orthodontists’ reports, there is no comprehensive organization and immediate access to the data of the patients’ orthodontic records, what inevitably jeopardize the defense of the orthodontist in legal compensation actions.

### Follow-up of treatment phases

There were frequent complains of the orthodontists concerning cooperation, especially from young patients, during the several phases of treatment. However, rarely the interviewees maintained control mechanisms such as patients’ absences. Equally rare are the cases in which there is organized control over the written awareness of patients or legal representatives during the whole treatment, as well as information and precautions about new therapeutic indications. Organized annotations related to technical opinions from other professionals involved in the treatment are also scarce. 

### Post-treatment follow-up

Post-treatment follow-up is even rarer, in times that consumers are eagerly following essential post-sale services in the acquisition of goods and services. This study showed that being careful to avoid future upsets, such as requiring a written consent of the patients at the end of treatment to keep addresses and contacts updated and making new appointments after treatment, are essential preventive measures that reveal good faith of the service provider. 

## DISCUSSION

Orthodontists are at risk of legal compensation actions that cannot be denied. Legal liabilities can lead to unpredictable and significant consequences for the continuity of their professional activity, producing adverse effects of personal, familial and social connotations that may compromise the future of their profession.^16^^,^^23^

There is no control over the will of the patient, because the activity of Orthodontics is ruled by the Consumer Defense Code, and thus, as a consumer, patients may postulate in court the occurrence of damage, in the broad sense of the term, that may result in an obligation of civil compensation.^24^^-^^26^ The possibility of claiming, by dissatisfied patients, may lead to unfavorable decision by the court, leaving the orthodontists no other option than to be prepared for the adequate exercise of the professional activity.^27^

The findings of this study demonstrated that a failure in the patient-professional relationship was identified in all groups of interviewees as a triggering element in judicial demands. It becomes mandatory, therefore, that the orthodontist should not neglect caution indicators and precautions in dealing and maintaining a good patient-professional relationship, established on the basis of the good faith principle. A standardized and customized service provision contract, oriented by a specialized lawyer, associated with available organized and complete orthodontic records, may constitute essential instruments for the orthodontists’ defense. On-demand resolutions, especially in the reasoning of a judicial decision, are the expert and documental evidences. The latter manifests itself in different ways, from the making of an adequate contract to the ordered maintenance of orthodontic records.

It should be taken into account the suggestions made by Rodrigues et al^9^ that the patient-professional contract constitutes a valuable instrument to protect those involved in the agreed clauses. Such contract should also be associated with a relationship based on loyalty, respect, and ethics.^26^ According to Guglinkski,^28^ this instrument should be characterized by the equilibrium of the contractual relationships.

Since deficiencies in the formation of orthodontists, as well as deficiencies in education concerning the juridical reflexes deriving from professional actions were also relevant aspects of the professional vulnerability, keeping updated with the profession and having a reasonable knowledge over juridical consequences of the exercise of orthodontics may minimize the risks of possible malpractice suits. 

This study demonstrated that there were failures in the formation, capacitation, and treatment follow-up, creating imprudent and negligent behaviors capable of putting at severe risk the orthodontists, who should not count exclusively on luck before a judicial demand, whether by the goodwill of the judge or by the incompetence or unpreparedness of the patients or the professionals that help with their activities. 

The indicated concerns, surrounded by cautions, are capable of providing the orthodontists with full right of technical, factual, and juridical defense in eventual judicial demands of compensatory nature. An important reflection was presented by Barroso et al^21^ that the fact of the patient knowing that the orthodontist is an organized professional with excellent capacitation and technical education may, often, prevent the possibility of a malpractice suit.

On the assumption that the patient-orthodontist relationship is part of a service agreement ruled by the Consumer Defense Code, the orthodontist should take necessary caution for the exercise of his/her professional activity using the principle of defensive logic, to eventually be prepared to justify their correct and adequate professional conduct according to the best ethical criteria and technical expertise. 

## CONCLUSION

Through this study, it could be verified that orthodontists do not have adequate formation for the comprehensive understanding of the juridical consequences of the exercise of their professional activity. Orthodontists failures could be noted throughout the entire treatment process, including establishing contractual relationships, acquisition of organized orthodontic records and, above all, records and information to patients or legal representatives during all phases of treatment.
